# Coronavirus disease 2019, food security and maternal mental health in Ceará, Brazil: a repeated cross-sectional survey

**DOI:** 10.1017/S1368980021000628

**Published:** 2021-05

**Authors:** Hermano AL Rocha, Christopher R Sudfeld, Álvaro JM Leite, Sabrina GMO Rocha, Márcia MT Machado, Jocileide S Campos, Anamaria Ce Silva, Luciano L Correia

**Affiliations:** 1 Department of Global Health and Population, Harvard T. H. Chan School of Public Health, Boston, MA, USA; 2 Department of Maternal and Child Health, Federal University of Ceará, Fortaleza, Brazil; 3 Department of Community Health, Federal University of Ceará, Fortaleza, Brazil; 4 Service, Education and Community Integration, University Center Unichristus, Fortaleza, Brazil

**Keywords:** COVID-19, Food supply, Mental disorders, Unemployment

## Abstract

**Objective:**

To quantify the change in the risk of food insecurity and maternal mental disorder (MMD) before and during the coronavirus disease 2019 (COVID-19) pandemic.

**Design:**

Repeated cross-sectional survey. Between 17 July and 10 September 2020, mother–child pairs who were enrolled in a population-based survey in 2017 were re-contacted by telephone for consent and to complete a telephonic COVID-19 survey. We used the Brazilian Food Insecurity Scale to assess food security and the Self Reporting Questionnaire-20 to assess MMD. McNemar’s test for paired data that also accounted for clustering was used. Logistic regression was used to assess the relationship of unemployment and receipt of government assistance with food insecurity and MMD in 2020.

**Setting:**

Ceará, Brazil.

**Participants:**

Five hundred and seventy-seven mother–child pairs completed the 2017 and 2020 surveys. At the time of the 2020 interview, the child cohort was 36–108 months of age.

**Results:**

The proportion of mothers reporting food insecurity was 15·5 % higher (95 % CI 5·9, 25·1, *P* value < 0·001) during the pandemic in July–August 2020 as compared with November 2017, while the prevalence of MMD was 40·2 % higher during the pandemic (95 % CI 32·6, 47·8, *P* value < 0·001). Loss of formal employment was associated with increased risk of food insecurity, but not with the risk of MMD.

**Conclusions:**

The risk of food insecurity and MMD in Ceará increased during the COVID-19 pandemic. These findings highlight the need for policies and interventions to reduce the impact of the COVID-19 pandemic on maternal and child health, nutrition and well-being in Brazil.

The global pandemic of coronavirus disease 2019 (COVID-19) has surpassed 48 million cases worldwide in November 2020, and Brazil has recorded more than five million confirmed cases^(1)^. Aiming to mitigate COVID-19 transmission, social distancing measures were implemented in many cities in Brazil, which led to isolation, school closures and unemployment, as well as potentially increased the risk of food insecurity and negatively impacted mental health and well-being^(2,3)^. Social distancing and school closures were implemented in Ceará, following the government lockdown decree on 19 March 2020. The Brazilian government provided COVID-19 assistance starting in April 2020. This assistance was a monthly cash payment of ˜US$120 for self-employed and informal workers over 18 years of age who do not receive any other benefit from the Federal Government (except for the conditional cash transfer programme), individuals who did not have formal employment and for families with a per capita monthly income (per person) up to half the minimum wage (about US$93) or a total monthly family income of up to three times minimum wage (about US$560).

The COVID-19 pandemic and related lockdowns may significantly increase the risk of food insecurity through multiple pathways including loss of employment and disruption of food supply chains^(4,5)^. A study conducted in Bangladesh found that the percentage of households experiencing food insecurity increased by 52 % during a COVID-19 lockdown as compared with 1–2 years before the pandemic^(6)^. In the USA, the COVID-19 Impact Survey found that 34·4 % of households with children ≤12 years old were food insecure in April 2020, compared with 15·1 % in 2018^(7)^. Further, multiple studies have found that the COVID-19 pandemic and lockdowns have negatively affected mental health in diverse contexts and populations^(8,9)^. Nevertheless, evidence on food insecurity and mental health in LMIC, particularly in Latin America, that compares rates before and during the pandemic is limited. To address this evidence gap, we report the change in the prevalence of food insecurity and maternal mental disorder (MMD) before and during the COVID-19 pandemic in Ceará, Brazil.

## Methods

We analysed data from the Maternal and Child Health Research in Ceará (*Pesquisa de Saúde Materno Infantil no Ceará* - PESMIC), a population-representative repeated cross-sectional survey focused on maternal and child health including children living in the state of Ceará, in northeastern Brazil. Ceará is the sixth poorest state in Brazil, with an average per capita income of US$150, and subsistence agriculture predominates in the rural area of the state. The PESMIC completed a survey round of mothers and children in 2017, and the full details of the methods can be found elsewhere^(10)^. Briefly, PESMIC 2017 survey round used random cluster based on the Brazilian Institute of Geography and Statistics census tracks. In each census track, twenty houses were randomly selected and households with children <72 months of age were eligible for enrolment. In 2020, eligible participants in the COVID-19 impact study were all 3566 mothers of children who were enrolled in the PESMIC survey round in 2017. Telephone contact number was available for all participants. Between 17 July and 10 September 2020, participants were re-contacted by trained interviewers by telephone for consent and to complete a survey. At the time of the 2020 interview, the child cohort was 36–108 months of age.

The study questionnaires in 2017 and 2020 included the Brazilian Food Insecurity Scale (*Escala Brasileira de Insegurança Alimentar* – EBIA), which is validated in Brazil for food insecurity screening^(11)^. Both survey rounds used the reduced version of EBIA, which included five question, and food insecurity was defined by the endorsement of any of the five questions. We did not use the full fifteen-question EBIA, and we were therefore not able to assess the severity of food insecurity. The Self Reporting Questionnaire-20 was also administered to assess symptoms of common mental disorders; MMD was defined as a score higher than 8 as per the scoring standard^(11,12)^. Employment status and receipt of government assistance were assessed by maternal self-report.

We examined the proportion of participants reporting food insecurity and MMD during 2017 *v*. the COVID-19 period in 2020 with McNemar’s test for paired data that also accounted for clustering by census track due to the design of the PESMIC study. We presented difference in proportions of food insecurity and MMD during the pandemic in 2020 as compared with 2017 with CI. Participants who were missing food insecurity (*n* 66) or MMD (*n* 83) data in 2017 were excluded from the analysis; all participants had complete data for 2020. We also examined the association of maternal employment and receipt of government assistance with food insecurity and MMD within the 2020 survey with logistic regression. We used separate univariate models with food insecurity and MDD as dependent variables and maternal employment and receipt of government assistance as independent variables (both were not included in same model). No participants were missing dependent or independent variables in the 2020 survey. Both survey rounds received approval from the Brazilian research ethics committee.

## Results

A total of 577 maternal–child pairs participated in the 2020 survey during the COVID-19 pandemic. The participants who completed the 2020 survey were relatively similar to those who were included in the 2017 PESMIC but were not followed up in 2020 (*n* 2989). Participants who were followed up had relatively similar risk of food insecurity as compared with those not followed up (55·0 % *v*. 60·1 %, *P* value = 0·122) and had slightly higher monthly income (R$ 1387·21 *v*. R$ 1040·53, a difference of ˜60 dollars/month, *P* value = 0·001).

In July–September 2020, during the COVID-19 pandemic, 68·9 % of the families reported food insecurity and 57·8 % of the mothers had Self Reporting Questionnaire-20 scores consistent with MMD, as compared with 54·4 % and 17·6 % before the pandemic in 2017, respectively. The difference in proportion of food insecurity was 15·5 % higher (95 % CI 5·9, 25·1, *P* value < 0·001) during the pandemic in July–August 2020 as compared with November 2017, while MMD was 40·2 % higher for MMD during the pandemic (95 % CI 32·6, 47·8, *P* value < 0·001) (Table 1).

**Table 1:** Food insecurity and maternal mental disorder for participants of the *Pesquisa de Saúde Materno Infantil no Ceará* survey in 2017 and 2020

2017 before COVID-19 pandemic	2020 during COVID-19 pandemic						Difference in proportions*	95 % CI	*P* value*
	Present		Absent		Total				
	Mean	sd	Mean	sd	Mean	sd			
Food insecurity									
Present	222	43·4	57	11·2	279	54·6	15·5 %	5·9, 25·1	<0·0001
Absent	136	26·6	96	18·8	232	45·4			
Total	358	70·1	153	29·9	511	100			
Maternal mental disorder									
Present	60	12·2	27	5·5	87	17·6	40·2 %	32·6, 47·8	<0·0001
Absent	225	45·6	181	36·7	406	82·4			
Total	285	57·8	208	42·2	493	100			

COVID-19, coronavirus disease 2019.

* Difference in proportion and *P* value from McNemar’s test that also accounted for cluster design.

Table 2 presents the relationship of unemployment and receipt of government aid with risk of food insecurity and MMD in 2020. About 62 % of the mothers who worked before the pandemic reported losing employment, and 69 % received the government COVID-19-related financial aid. Loss of formal employment was associated with increased likelihood of food insecurity (OR: 6·70; 95 % CI 3·14, 14·30). In addition, families that received government assistance also had increased likelihood of food insecurity (*P*-values < 0·001). Unemployment and receipt of government assistance were not associated with MMD in 2020 (Table 2).

**Table 2:** Association of maternal unemployment and receipt of government coronavirus disease 2019 (COVID-19) financial aid with food insecurity and maternal mental disorder (MMD) in 2020 (*n* 577)

	Food insecurity during COVID-19 pandemic					MMD during COVID-19 pandemic				
	*n*/*N*	%	OR	95 % CI	*P* value	*n*/*N*	%	OR	95 % CI	*P* value
Maternal unemployment due to COVID19					<0·001					0·11
No, did not work before pandemic	204/275	73·0	2·37	1·50, 3·74		170/275	61·8	1·59	1·04, 2·44	
Yes, lost formal job	99/116	85·3	6·70	3·14, 14·30		69/116	59·5	1·34	0·77, 2·31	
Yes, lost informal job	14/19	73·7	2·41	0·73, 7·97		13/19	68·4	2·67	0·81, 8·84	
No, currently employed	90/167	53·9	Reference			87/167	52·1	Reference		
Family received government’s financial aid					<0·001					0·93
No	99/173	57·2	0·38	0·25, 0·59		99/173	57·2	0·98	0·65, 1·46	
Yes	308/404	76·2	Reference		240/404	59·4	Reference			

## Discussion

In this population-based cohort in Ceará, Brazil, we found that the prevalence of food insecurity increased by 15·5 % during the COVID-19 and MMD increased by 40·2 % from 2017 to 2020 during the COVID-19 pandemic. Our results also suggest that during 2020, loss of employment and receipt of government aid (an indicator of poverty) were associated with an increased risk of food insecurity.

Increased risk of food insecurity during the COVID-19 pandemic is in line with studies in high-income and LMIC settings^(6,7)^. We found that formal job loss was markedly associated with increased risk of food insecurity. Ceará has been economically stable since 2017; therefore, job losses reported in the 2020 survey were most likely related to the COVID-19 pandemic. In addition to unemployment, the interruption of food supply chains can also contribute to the increased risk of food insecurity^(13)^; however, in Ceará, there were no major interruptions in the distribution of food reported during the study period.

In addition, Pfefferbaum *et al.* suggested that COVID-19 and public health actions like social distancing may negatively impact mental health through isolation, loss of income and uncertainty about the future^(14)^. In this study, MMD significantly increased during the pandemic; however, job loss and the need for government assistance were not associated with increased risk and therefore suggest that the other factors negatively contributed to poorer mental health. Of note, food insecurity is associated with poorer mental health and may be a contributor in our study^(15)^. Food insecurity and MMD can negatively impact child nutritional status, parenting and child development and represent an unprecedented risk to children’s health and well-being^(16–21)^.

Our study has several limitations. First, our study may be at risk of attrition bias as not all participants surveyed in 2017 were followed up in 2020; however, we found that participants who were followed up were relatively similar to those not followed up. In addition, the difference in the modality of data collection between survey rounds (in-person *v*. telephone) may have led to information bias; however, it is not clear the degree to which participants may over- or underreport food insecurity and MMD by telephone.

Ceará is one of the poorest states of Brazil, and the average monthly per capita income of US$ 150 can be compared with that of many other developing countries. These findings highlight the need for policies and interventions to reduce the impact of the COVID-19 pandemic on food insecurity and maternal mental health in Brazil. A few potential strategies include extending or increasing the government COVID-19 assistance programme, direct provision of food to families in need and expanded access to mental health services.
